# Questionnaire and analysis of the standardized use of home self-monitoring portable blood glucose meters

**DOI:** 10.1097/MD.0000000000041330

**Published:** 2025-01-24

**Authors:** Hongrui Zhang, Lu Cheng, Meng Li, Xiongwei Ye, Xiaochen Wan

**Affiliations:** a Department of Clinical Laboratory, Zhejiang Hospital, Hangzhou, Zhejiang, China.

**Keywords:** awareness rate, diabetes, portable blood glucose meters, questionnaire, self-monitoring blood glucose

## Abstract

To evaluate the accuracy of home self-monitoring portable blood glucose meters, we analyzed the current problems of patients using portable blood glucose meters and put forward reasonable suggestions. A self-designed questionnaire was used to survey 142 patients and 132 healthcare professionals. The questionnaire consisted of 16 items with an overall score ranging from 1 to 13 (with a higher score indicating better experience). The study included adult patients with diabetes mellitus type 1 or type 2. The patients’ awareness rate for standardized use of portable blood glucose meters at home was at 31.42%. Specifically, awareness of blood collection specifications was at 15.85%, standardized operation at 42.53%, and accuracy of results at 0%. A significant majority (81.69%) scored between 0 and 5 points in home blood glucose meter use, with knowledge and practice scores consistently below 3 points. Notably, no patients conducted a formal comparison of various meters. Despite this, their confidence level in the portable blood glucose meter results was as high as 71.1%. Urgent measures are needed to educate patients on proper usage and encourage regular instrument comparisons.

## 1. Introduction

According to the World Diabetes Map (8th Edition) by the International Diabetes Federation, approximately 425 million adults over the age of 20 worldwide suffer from diabetes.^[1]^ China has the highest number of diabetic patients. Currently, there is no cure for diabetes, and long-term treatment is necessary to maintain blood glucose levels. If early glycemic control is poor, persistent hyperglycemia may lead to a series of pathological changes that continue to accumulate and “memorize,” and even if blood glucose is controlled to some degree later in life, previous adverse metabolic effects may remain, increasing the likelihood of the development of chronic complications of diabetes mellitus.^[2]^ Early glycemic control may have a long-lasting beneficial effect, reducing the risk of serious micro- and macro-vascular complications, known as the legacy effect or metabolic memory.^[3]^ Portable blood glucose meters allow patients to monitor their own blood glucose levels from time to time, facilitating early control of their own blood glucose and greatly avoiding the metabolic memory effect described above.

Self-monitoring blood glucose (SMBG) can assist patients with diabetes to better understand their glycemic status, and consequently to adopt appropriate actions to cope with hyper- or hypoglycemia. SMBG has been shown to improve glycemic control among patients with diabetes.^[4–6]^ Furthermore, a home self-monitoring portable blood glucose meter is an important means to ensure SMBG. In recent years, more and more diabetic patients need continuous glucose monitoring to understand their blood glucose changes in real time, especially for type 1 diabetic patients and type 2 diabetic patients with large fluctuations in blood glucose, and its application has become increasingly popular. Portable glucose meters, with their superior monitoring accuracy and stability, as well as data storage and analysis functions, have greatly catered to this need of diabetic patients.

There are many challenges associated with the use of portable blood glucose meters, which are mainly related to quality assurance.^[7]^ The management of home self-monitoring portable blood glucose meters is performed by patients at home rather than trained individuals in the laboratory, which can lead to errors resulting from the lack of understanding the importance of quality control and quality assurance practices. Most of the current research concerns the investigation of blood glucose monitoring systems (BGMS) used in medical institutions, and there is still a lack of studies on patients’ awareness of the accuracy of home self-monitoring portable blood glucose meters and the standardized use of home blood glucose meters.^[7–9]^

In this study, a questionnaire survey was employed to investigate the awareness of the standardized use of home self-monitoring portable blood glucose meters in outpatient and inpatient diabetic patients in our hospital. Combined with the author’s several years of clinical work and management experience of blood glucose meters, this paper analyzes the current problems of patients using portable blood glucose meters and puts forward reasonable suggestions for the management of home self-monitoring portable blood glucose meters for diabetic patients.

## 2. Methods

### 2.1. General information

During the period of November 2019 to September 2021, questionnaires were administered to 142 patients and 132 healthcare professionals in Zhejiang Hospital, respectively. The inclusion criteria of patients were as follows: (1) aged ≥ 18 years; (2) patients with type 1 diabetes or type 2 diabetes and using insulin; (3) patients using blood glucose monitoring equipment; (4) no impairment in language communication and comprehension. The exclusion criteria were: (1) patients with mental illness who cannot cooperate; (2) pregnant and lactating patients. Healthcare professionals inclusion criteria: randomized questionnaires for all hospital physicians. Before the investigation, patients were informed of the purpose of the study and the contents to be filled in, and the patients and healthcare professionals participated voluntarily and filled in the questionnaires anonymously. This study has been approved by the Scientific Research Ethics Committee of Zhejiang Hospital. The data collection period of this study: from November 2019 to September 2021. The sample size is 274, taking into account the human, resource and financial resources and the degree of tolerance of error in the study, the sample size determined in this study is calculated as: N = Z^2^ × (P × (1-P))/E^2^, where N is the sample size and Z is the statistic; Z = 1.96 when the confidence level is 95%; Z = 1.64 when the confidence level is 90%; E is the error value and P is the probability value. The sample included both patients and healthcare professionals with multiple types of diabetes and was representative of a broader group of patients and healthcare professionals with diabetes.

### 2.2. Questionnaire design

The survey was conducted by using 2 self-made questionnaires. Questionnaire 1 was on the standardized use of home self-monitoring portable blood glucose meters, including questions on the general situation of patients and those on the knowledge, attitude and behavior of self-monitoring of blood glucose. The general information questionnaire of patients included the patient’s age, years of diabetes, years of use of portable blood glucose meters, and the brand of portable blood glucose meters. The main part of the patient’s self-glucose monitoring knowledge, attitude and behavior questionnaire was divided into 3 parts: standardized operation of blood collection, standardized operation of blood glucose meter, and accuracy of blood glucose meter results. The questionnaire contained a total of 16 items, as shown in Table S1, Supplemental Digital Content, http://links.lww.com/MD/O295.

Questionnaire 2 was on the standardized use of home self-monitoring portable blood glucose meters by healthcare professionals, including questions on the general situation of medical care and those on the knowledge, attitude, and behavior of self-monitoring blood glucose. The questionnaire on general medical conditions included age, years of work, nature of work, and whether the patient is a healthcare professionals member in the department of endocrinology. The main part was on the standardized operation of blood collection, standardized operation of blood glucose meter, and accuracy of blood glucose meter results, which contained 13 items as shown in Table S2, Supplemental Digital Content, http://links.lww.com/MD/O295.

A positive answer of “yes” to a single choice question was worth 1 point, while a “no” answer was worth 0 points, with points accumulating with the choices. The pre-survey questionnaire had a total of 16 (13) items, and the sample size was calculated as 5 to 10 times the number of items. The sample size of the survey should be 80 to 160 (65–135) cases, considering the possibility of lost follow-up and invalid samples. We increased the sample size by 20% on the original basis, that is, the theoretically required sample size was 96 to 192 (68–162) cases.

In this study, the questionnaire was validated using expert review to ensure the reliability of the questionnaire and to minimize the level of bias. We selected a group of 10 professionals, all holding senior titles or higher and with more than 20 years of experience in their respective fields. This group includes 2 experts in medical engineering, 3 chief physicians specializing in endocrinology, 2 senior nursing specialists with chief titles, and 3 chief physicians specializing in laboratory medicine. To assess the content validity, the experts were asked to evaluate the relevance, clarity, and representativeness of each item in the questionnaire. Each expert provided ratings for each question on a 4-point scale (1 = Not relevant, 4 = Highly relevant). After collecting the experts’ feedback, content validity ratio (CVR) was calculated for each item, using the formula: CVR = (Ne − N/2)/(N/2), where Ne is the number of experts who rated the item as relevant, and N is the total number of experts. The average CVR across all items was calculated to assess overall content validity. To assess the consistency of the experts’ evaluations, inter-rater reliability was calculated. Each expert independently rated the items on the same 4-point scale. Intraclass Correlation Coefficient was used to determine the level of agreement between raters. After several rounds of evaluation and feedback from experts, we made several revisions to the questionnaire and finalized the final version. The content validity of the questionnaire in this study was 0.83 and the inter-rater reliability was 0.85.

### 2.3. Questionnaire evaluation

According to the results recovered from the questionnaire, the patients’ awareness rate of standardized use of home self-monitoring portable blood glucose meters was calculated according to the Guidelines for clinical operation and quality management of portable blood glucose meters.^[10]^ The full score for each patient was 13, and the full score for each healthcare professionals was 10. Among them, if the respondent performed any operation in the questionnaire in a standardized manner, the knowledge score was 1 point, whereas if the respondent did or did not know whether any operation was performed, the knowledge score was 0 points. The awareness rate of the standardized use of home self-monitoring portable blood glucose meters (%) was calculated as the respondents’ portable blood glucose meter use knowledge score/total scores of all respondents using portable blood glucose meters × 100%.

### 2.4. Statistical methods

All data were analyzed using SPSS 19.0 statistical software. Normally distributed measures were expressed as (x ± s), and non-normally distributed measures were expressed as median [M(P25, P75)]. The descriptive statistics were expressed as number of cases and percentages, and statistical inferences were made using the independent samples *t* test.

## 3. Results

### 3.1. General information

A total of 142 questionnaires from the diabetes subjects of patients were valid. The subjects were aged 18 to 100 (68.7 ± 13.7) years, and the duration of diabetes was 1 month to 30 years, with a median of 9 years. The useful life was 1 month to 25 years, with a median of 7 years.

A total of 132 healthcare professionals were included in this study, and 132 valid questionnaires were recovered. The age of 132 healthcare professionals was 25 to 55 years with a median of 41 years; their working years were 1 to 30 years with a median of 14 years, and 5 of them were endocrinology healthcare professionals.

### 3.2. Evaluation of the knowledge, belief and practice of the standardized use of home self-monitoring portable blood glucose meters

From the analysis of patients’ questionnaires, it was found that the awareness rate of standardized use of home self-monitoring portable blood glucose meters was 31.42%, among which the awareness rate of blood collection specifications was 15.85%, the awareness rate of standardized operation of blood glucose meters was 42.53%, and the awareness rate of the accuracy of blood glucose meter results was 0. The specific statistics are shown in Table 1. This may be due to the fact that patients have not received systematic and detailed training on blood collection operations and are unfamiliar with the correct steps and points of blood collection. In addition, patients may be unaware of the existence of a certain margin of error in the blood glucose meter itself, as well as of the impact of other factors such as improper storage of test strips and environmental disturbances on accuracy.

**Table 1:** Results for the standardized use of home portable blood glucose meters.

Questionnaire items	Number of people scoring (percentage)
Appropriate amount of blood	30 (21.13%)
75% alcohol disinfection, blood collection after drying	24 (16.90%)
Do not use iodophor	26 (18.31%)
Do not squeeze hard after wiping off the first drop of blood after blood collection	10 (7.04%)
Correct operation	50 (35.21%)
Matching test paper	126 (88.73%)
Instrument calibration	41 (28.87%)
Test strips should be stored dry and within the validity period	49 (34.51%)
Regular maintenance	36 (25.35%)
Awareness rate of result accuracy	0 (0%)
Regular comparison has been done	0 (0%)
Irregular instrument comparison has been done	87 (61.3%)
Confidence in blood glucose meter results	101 (71.1%)

**Figure 1.**
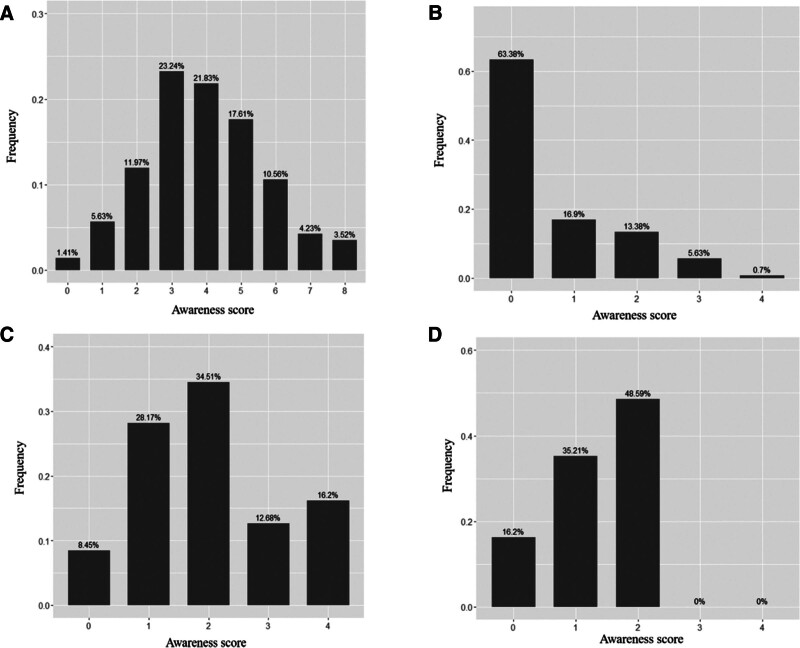
The awareness score in patients. (A) Scores of the questionnaire “Knowledge, Belief, and Action”; (B) scores of blood collection standard practice; (C) scores of glucose meter standardized operation; (D) scores of blood glucose meter results.

According to the analysis of the results for the standardized use of home portable blood glucose meter score table for knowledge, belief and behavior, as high as 81.69% of patients had a total score between 0 and 5 points on the standardized use of knowledge, belief and behavior of home portable blood glucose meters, as shown in Figure 1A. The reason may be that some patients think that portable blood glucose meter operation is simple and do not pay enough attention to it, and operate it randomly based on self-feeling, resulting in improper operation.

### 3.3. Patients’ knowledge of blood collection practices

By analyzing the scores of the standardized operation of blood collection (collection of an appropriate amount of blood, 75% alcohol disinfection, blood collection after drying, avoiding the use of iodophor, wiping off the first drop of blood after blood collection while not squeezing hard), blood was collected from up to 63.38% of patients. The standardized operation score was 0 points, indicating that no patient performed a completely correct blood collection operation, and incorrect blood collection practices were most likely to directly lead to flawed test results, as detailed in Figure 1B.

### 3.4. Awareness rate of the standardized operation of blood glucose meters in patients

When comparing the patients’ glucose meter normative operation scores (correct operation, matching test strips, test strips stored dry and used within the validity period, instrument calibration, and regular maintenance), 71.13% of patients scored 0 to 2 points, indicating that most patients rarely understood the operation of blood glucose meters, most of them had problems with the operation, and no patient correctly grasped the operating specifications of the blood glucose meter, as detailed in Figure 1C.

### 3.5. Patients’ awareness rate of blood glucose meter results

According to the statistics for patients’ glucose meter result scores (result accuracy awareness rate, regular comparison, irregular instrument comparison, degree of reliability of blood glucose meter results), patients’ knowledge, belief and behavior scores were all <3 points, and patients’ understanding of blood glucose meter results was generally low. Their confidence level of the portable blood glucose meter results was as high as 71.1%. It is difficult to make a correct judgment on the accuracy and significance of the results; see Figure 1D for details. The reason for this may be related to the lack of physicians or professionals to educate and remind them about accuracy.

### 3.6. Self-assessment of the standardized use of home portable blood glucose meters by healthcare professionals

By comparison, 89.39% of the healthcare professionals knew that a blood glucose meter should be compared regularly, but when the healthcare professionals checked the patient’s blood glucose monitoring record, it was revealed that only 64.39% of the doctors would ask the patient about the blood glucose meter; in addition, the knowledge about standardized blood collection was also low among healthcare professionals. It could be seen that healthcare professionals were not familiar with the relevant knowledge on the correct use of blood glucose meters, and had a lack of awareness of publicity. The detailed results are shown in Table 2.

**Table 2:** Statistics on the results for the questionnaire filled by healthcare professionals.

Classification	Yes (percentage/%)
Appropriate amount of blood	98 (74.24)
75% alcohol disinfection, blood collection after drying	98 (74.24)
Do not use iodophor	92 (69.7)
Correct operation	105 (79.55)
Matching test paper	130 (98.48)
Instrument calibration	118 (89.39)
Test strips should be stored dry and within the validity period	116 (87.88)
Regular maintenance	124 (93.94)
Whether the healthcare professionals will ask the blood glucose meter status when viewing the patient’s blood glucose monitoring record	85 (64.39)
Whether they will teach the patient how to use the portable blood glucose meter correctly	107 (81.06)

**Figure 2.**
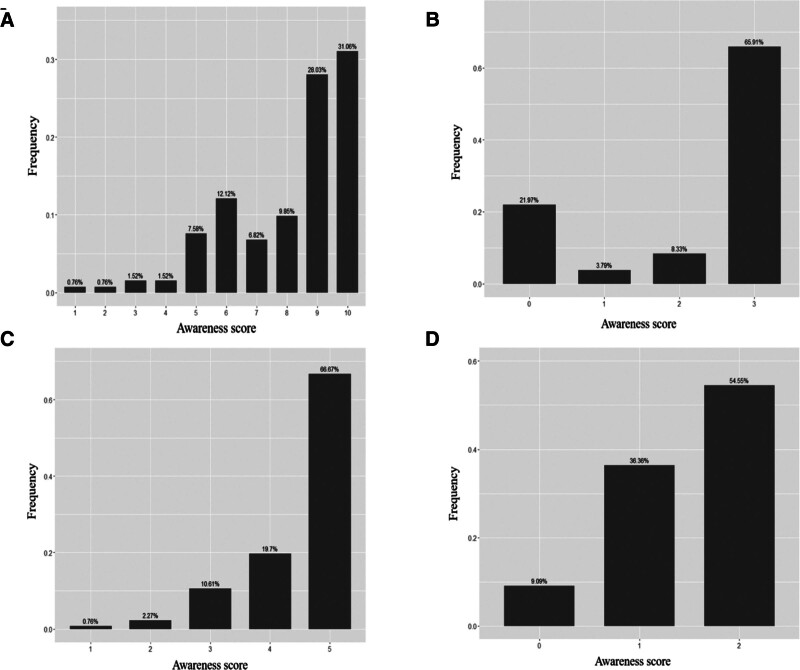
The awareness score in healthcare professionals. (A) Scores of the questionnaire “Knowledge, Belief, and Action”; (B) standardized operation score of blood collection; (C) standardized operation score of blood glucose meter; (D) medical and nursing staff missionary status score.

According to the analysis of the results for the knowledge, belief and behavioral score table for the standardized use of portable blood glucose meters by healthcare professionals at home, the scores (the final score for all questions) totaled 8 to 10 points. That is, most health care workers had a certain understanding of the standardized use of portable blood glucose meters, while some of them lacked the relevant knowledge. The specific statistics are shown in Figure 2A.

The analysis of the standardized operation scores of blood collection for healthcare professionals (appropriate amount of blood collection, 75% alcohol disinfection, blood collection after drying, do not use iodophor) showed that only 65.91% of healthcare professionals had blood collection operations that met the requirements, and 21.97% of the health care workers had a complete lack of knowledge of the standard practice of blood collection (see Fig. 2B).

As for the score of standardized operation of blood glucose meters for healthcare professionals (correct operation, matching test strips, test strips stored dry and within the validity period, instrument calibration, regular maintenance), the survey showed that the standardized operation score for blood glucose meters was 5 points for only 66.67% of healthcare professionals. Meanwhile, 48.48% of healthcare professionals did not have a correct and comprehensive understanding of the operating specifications of the blood glucose meter, and as a consequence, irregular operation affected the accuracy of the results (see Fig. 2C for details).

According to the analysis of medical and nursing staff missionary ratings (whether they would teach patients how to use the portable blood glucose meter correctly, and whether the healthcare professionals would ask about the blood glucose meter when viewing the patient’s blood glucose monitoring record), our survey found that only 64.39% of health care workers would ask about the glucose meters when reviewing patients’ glucose monitoring records, and 9.09% of healthcare professionals would not carry out publicity and education activities on patients, as shown in Figure 2D.

## 4. Discussion

Diabetes remains one of the most consequential chronic diseases worldwide considering the acute and chronic complications associated with poor glycemic control.^[11]^ To achieve the desired glycemic control targets, an active attitude in patients is needed, and SMBG plays a significant role. Nowadays, studies have highlighted that SMBG has been shown to reduce hemoglobin A1C.^[5,12]^ Furthermore, home self-monitoring portable blood glucose meters are important means to ensure SMBG.

While these developments have advanced diabetes care, they also present issues associated with the clinical effects of accuracy, and errors in portable blood glucose meters as well as user competency.^[9,13]^

The American Diabetes Association recognized that a major source of variability for glucose meter results was user competency.^[14]^ The survey results showed that the scores of knowledge, belief and behavior (the final score for all questions) for the standardized use of portable blood glucose meters in patients at home were mainly between 0 and 5 points, and most of them had problems such as irregular blood collection and irregular operation of blood glucose meters. The awareness rate of blood collection specifications was only 15.85%, and the awareness rate of standardized operation of blood glucose meter was only 42.53%. The comparison of home portable blood glucose meters with core lab testing revealed a lack of competency during blood collection and handling results in higher pre-analytical error rates for the former.^[15]^ This bias can be reduced by focusing on quality management efforts in pre-analytical processes.^[16,17]^ This may be due to the fact that diabetic patients and healthcare professionals do not receive comprehensive and in-depth training and guidance on the use of blood glucose meters, among other things. They may not be motivated to seek out and learn about the subject, and may be psychologically overly reliant on automated technology, not keeping up to date with new advances and requirements in the field of blood glucose meters.

In addition, people in different economic levels may have different levels of awareness and importance of the disease. In economically developed regions, people may have a higher ability to pay for portable glucose meters and a relatively well-developed healthcare system that is more conducive to their widespread adoption, whereas in less economically developed regions, cost issues may limit their popularity. Differences in social class may also be reflected, with higher class populations likely to have more resources and awareness of its use. The distribution of healthcare resources in a region will also have an impact, with resource-rich places better able to promote and guide standardized use. The level of development of a society also determines the rate of acceptance of health technology; developed societies may be more likely to incorporate portable glucose meters into routine health management. Differences in educational level may lead to different levels of understanding and mastery of the instructions for using the instruments, which in turn may affect standardized use. The degree of importance and investment in chronic disease management by society will also influence the promotion and implementation of standardization of its use.

The application of artificial intelligence may be a potential solution to this problem. By applying AI drivers, we can customize the personalized reminder time and frequency for each patient based on their daily habits, blood glucose fluctuation patterns, and so on, to ensure that they don’t forget to test their blood glucose. In addition, timely reminder messages can be sent, which can effectively overcome patients’ inertia and negligence, and cultivate the habit of regular testing. When irregularities in operation may occur during the testing process, such as improper blood collection, the AI program can immediately remind to correct and avoid wrong results.

The International Federation for Clinical Chemistry and Lab Medicine,^[11]^ American Association for Clinical Chemistry,^[18]^ and Med LAB Observer^[19]^ have published dozens of management regulations to increase the quality assurance of BGMS in medical institutions. As a result, this study found that the scores of knowledge, belief and behavior in the standardized use of portable blood glucose meters by healthcare professionals were mainly in the range of 8 to 10 points, which was significantly higher than the score of patients. However, these papers only controlled for the hospital or laboratory, and specifically excluded testing performed by patients.^[10,20]^ SMBG has always been in an unsupervised state^[9]^; therefore, it is necessary to strengthen publicity and education on the operation of blood collection and blood glucose meter for patients. In addition, it is urgent to build standardized guidelines to ensure that patients perform blood glucose self-testing in accordance with unified operating procedures.

Moreover, due to deviations in the environmental temperature, humidity, maintenance, and performance of the blood glucose meter itself, each link will directly affect the test results of the portable blood glucose meter, resulting in a large error in the blood glucose results.^[21]^ Through the analysis of the patients’ questionnaires, it was found that the awareness rate of the accuracy of the results of the portable blood glucose meter was 0, indicating that patients had a serious lack of awareness of the accuracy of the blood glucose meter results. However, patients’ trust in these results was as high as 71.1%, which indicates that patients completely ignored the performance error of the blood glucose meter itself and blindly trusted the test results. This is in line with the findings of James Dalton,^[9]^ who stated that patients would be highly confident in that the results of home portable blood glucose meters are “accurate.” In contrast to the accuracy and precision of glucose meters under controlled laboratory conditions where CVs were usually 2% to 5%, up to 50% of the SMBG results could vary more than 20% from a reference value for meters in general use.^[14]^

Therefore, the regular comparison and quality control of home portable blood glucose meters is of great significance to ensure the accuracy of blood glucose data. The Guidelines for Clinical Operation and Quality Management of Portable Blood Glucose Meters^[10]^ stipulates that the comparison and evaluation of the results of portable blood glucose meters and large biochemical tests should be performed no less than once every 6 months. The reliability of data from every blood glucose meter in the laboratory is guaranteed by standard quality control and calibration. On the other hand, this survey showed that no patients were aware of the need for regular comparisons with home portable blood glucose meters, and such instruments do not have quality control procedures to satisfy the patient’s self-checking of the instrument. To address this serious deficiency, it is necessary to popularize the knowledge of home self-monitoring portable blood glucose meters. For example, the relevant institutions can provide services for the calibration of blood glucose meters for patients, and the manufacturer of blood glucose meters should also provide patients with a quality control protocol to provide more comprehensive quality assurance.

In addition, the National Diabetes Association can ensure that healthcare professionals are proficient in the content of the guidelines and the correct way to operate them by organizing training courses and seminars for them. Educational activities are also conducted for patients and their families to let them know how to use blood glucose meters correctly and the precautions to be taken. A blood glucose meter quality monitoring system has also been established to regularly test and evaluate blood glucose meter products in the market to ensure that they meet the standards. Supervision and inspection of the use of blood glucose meters in medical institutions should be carried out, and problems should be corrected and rectified in a timely manner. In addition, compliance with the guidelines on the use of portable blood glucose meters can be incorporated into the assessment indicators of medical institutions, prompting them to pay attention to and strictly implement them. All of the above measures will be conducive to the promotion of standardized operation of portable blood glucose meters.

Healthcare professionals can have an important role in improving the quality assurance of home portable glucose meters.^[9]^ By analyzing the questionnaires from healthcare professionals, it was found that 89.39% of them were aware that portable blood glucose meters should be compared regularly; meanwhile, when healthcare professionals checked patients’ blood glucose monitoring records, only 64.39% of doctors would ask patients about their blood glucose meters. From these results, it can be seen that health care personnel were not familiar with the knowledge related to the correct use of portable blood glucose meters. Besides, the awareness of propaganda is insufficient among health care personnel, which is also one of the important reasons for the low score of patients on the correct use of blood glucose meters. Thus, the awareness of propaganda among health care personnel should be strengthened.

This study analyzed the standardized use of home self-monitoring portable blood glucose meters through a questionnaire. The questionnaire was validated using expert review to ensure its reliability and minimize potential biases. However, it is important to note that while expert review can reduce content-related biases, it does not inherently address other types of bias, such as response bias, social desirability bias, or sampling bias.

## 5. Conclusions

The present study revealed that there are problems in the cognition and operation of portable blood glucose meters by both patients and healthcare professionals, especially in terms of the accuracy of such instruments and user competency, which lead to the inability of users to judge the accuracy of the results provided by these instruments. One of the best strategies to address this problem should be to first educate patients on the use of home portable blood glucose meters, and to promote the regular comparison service of portable blood glucose meters at home. The greatest positive effects are likely to result from the wide dissemination of knowledge content on the operation of portable blood glucose meters using multiple channels (e.g., professional journals, websites, social media, etc). This should be reinforced with comprehensive quality systems that monitor technical performance among other tasks. We are calling not only for hospitals to take action, but also for healthcare policymakers, blood glucose monitoring device manufacturers, and public health educators to work together to ensure the accuracy and effectiveness of home BGMS. The next step in focusing research on the accuracy of glucose meters used by people with diabetes would be ideal for assessing progress in implementing relevant measures.

## Author contributions

**Conceptualization:** Hongrui Zhang, Xiaochen Wan.

**Data curation:** Hongrui Zhang, Lu Cheng, Xiongwei Ye.

**Investigation:** Hongrui Zhang, Lu Cheng, Xiongwei Ye.

**Writing – original draft:** Hongrui Zhang, Meng Li.

**Writing – review & editing:** Hongrui Zhang, Lu Cheng, Meng Li, Xiongwei Ye, Xiaochen Wan.

## Supplementary Material


