# Supplementary material for: Questionnaire and analysis of the standardized use of home self-monitoring portable blood glucose meters
Source: Medicine (Baltimore). 2025 Jan 24;104(4):e41330. doi: 10.1097/MD.0000000000041330 (PMC11771602; doi:10.1097/MD.0000000000041330)
Supplement: Supplementary file 1 [file medi-104-e41330-s001.docx]

Table S1. Questionnaire on the normative use of home self-monitoring portable blood glucose meters by patients

| **Classification** | **Specific project** |
| --- | --- |
| General information | Age  Years of illness  Blood Glucose Meter Use Period |
| Main content:  Standardized operation of blood collection  Standardized operation of blood glucose meter  Blood glucose meter results | 1. Collection of appropriate amount of blood  2.75% alcohol disinfection, blood collection after drying  3. Do not use iodophor  4. After wiping off the first drop of blood after blood collection, do not squeeze hard  1. Check whether the operation is done correctly  2. Matching test paper  3. The test strip should be stored dry and within the validity period  4. Instrument Calibration  5. Regular maintenance  1. Awareness rate of result accuracy  2. Performed regular comparison  3. Performed irregular instrument comparison  4. Confidence in blood glucose meter results |

Table S2. Questionnaire on the knowledge, belief and behavior of healthcare professionals on the standardized use of home self-monitoring portable blood glucose meters

| **Classification** | **Specific project** |
| --- | --- |
| General information | Age  Working years  Whether they are an endocrinologist |
| Main content:  Standardized operation of blood collection  Standardized operation of blood glucose meter  Educational situation of healthcare professionals | 1. Appropriate amount of blood collection  2.75% alcohol disinfection, blood collection after drying  3. Do not use iodophor  1. Correct operation  2. Matching test paper  3. The test strip should be stored dry and within the validity period  4. Instrument calibration  5. Regular maintenance  1. Whether the healthcare professionals will ask about the blood glucose meter when viewing the patient's blood glucose monitoring record  2. Whether they will teach the patient how to use the portable blood glucose meter correctly |
